# The Role of Polyunsaturated Fatty Acids (PUFAs) in the Primary Prevention of Allergic Diseases in Children: A Position Paper of the SIAIP Primary and Secondary Prevention of Allergic Diseases and Nutraceuticals Committees

**DOI:** 10.3390/nu18132072

**Published:** 2026-06-24

**Authors:** Angela Klain, Cristiana Indolfi, Giorgio Ciprandi, Alberto Martelli, Francesco Paolo Brunese, Salvatore Cascone, Valentina Cattivera, Lorenzo Cresta, Giulio Dinardo, Cecilia Fabiano, Filippo Favuzza, Francesca Galletta, Carolina Grella, Amelia Licari, Sara Manti, Antonio Andrea Senatore, Irene Schiavetti, Chiara Trincianti, Michele Miraglia del Giudice, Gianluigi Marseglia

**Affiliations:** 1 Department of Woman, Child and General and Specialized Surgery, University of Campania ‘Luigi Vanvitelli’, 80138 Naples, Italy; klainangela95@gmail.com (A.K.); cristianaind@hotmail.com (C.I.); salvatore.cascone2@gmail.com (S.C.); dinardogiulio@gmail.com (G.D.); caro.grella94@gmail.com (C.G.); michele.miragliadelgiudice@unicampania.it (M.M.d.G.); 2 Department of Medicine and Health Sciences, University of Molise, 86100 Campobasso, Italy; 3 School, Family, and Association Task Force, Italian Society of Pediatric Allergy and Immunology, 20126 Milan, Italy; 4 Primary Care Pediatrics, ASL Caserta, 81100 Caserta, Italy; francescopaolobrunese@gmail.com; 5 Department of Pediatrics, University of L’Aquila, 67100 L’Aquila, Italy; valecatt.96@gmail.com (V.C.); fabiano.cecilia@yahoo.it (C.F.); 6 Primary Care Pediatrics, ASL Genoa, 16100 Genoa, Italy; crestalorenzo1893@gmail.com; 7 Pediatric Unit, Hospital Holy Family Fatebenefratelli, 22036 Erba, Italy; filippofavuzza@virgilio.it; 8 Pediatric Unit, Department of Human Pathology in Adult and Developmental Age ‘Gaetano Barresi’, University of Messina, 98124 Messina, Italy; francygall.92@gmail.com (F.G.); sara.manti@unime.it (S.M.); 9 Pediatric Unit, Department of Clinical, Surgical, Diagnostic and Pediatric Sciences, University of Pavia, 27100 Pavia, Italy; amelia.licari@unipv.it (A.L.); gianluigi.marseglia@unipv.it (G.M.); 10 Pediatric Clinic, Fondazione IRCCS Policlinico, 27100 Pavia, Italy; antonioandrea.senatore01@universitadipavia.it; 11 Health Science Department, University of Genoa, 16100 Genoa, Italy; irene.schiavetti@unige.it; 12 UOSD Allergy Center, IRCCS Istituto Giannina Gaslini, 16100 Genoa, Italy; chiara.trincianti@gmail.com

**Keywords:** polyunsaturated fatty acids, PUFA, allergic diseases, primary prevention, children, omega-3

## Abstract

**Background:** Type 2 inflammatory diseases are among the most common chronic inflammatory conditions in childhood and represent a growing global health burden. Increasing evidence suggests that early-life nutritional exposures may influence immune programming and allergic disease development. This Position Paper aims to summarize the current evidence regarding the immunomodulatory role of polyunsaturated fatty acids (PUFAs), particularly omega-3 long-chain fatty acids, in the prevention of allergic diseases during early life. **Methods:** A scoping literature review and consensus process were conducted to map biological mechanisms and clinical evidence linking omega-3 PUFAs with allergic disease prevention. This document analyzed experimental, observational, and randomized controlled studies evaluating maternal prenatal/lactational omega-3 exposure. The clinical evidence was qualitatively appraised using study-design-specific Joanna Briggs Institute (JBI) Critical Appraisal Tools. Particular attention was given to immune modulation, inflammatory pathways, epithelial barrier function, gut microbiota interactions, and the ferroptosis–immune–metabolic axis. **Results:** Omega-3 PUFAs, including eicosapentaenoic acid (EPA) and docosahexaenoic acid (DHA), exert immunomodulatory and anti-inflammatory effects through multiple mechanisms, including specialized pro-resolving mediator production, regulation of T-helper cell responses, cytokine modulation, maintenance of epithelial barrier integrity, and microbiota interaction. Emerging evidence also supports their involvement in oxidative stress and ferroptosis regulation. Current clinical evidence, particularly from higher-quality prenatal randomized trials and evidence syntheses, suggests that adequate maternal omega-3 intake during pregnancy and lactation may reduce the risk of respiratory allergic outcomes, especially wheezing and asthma, in selected offspring. **Conclusions:** Adequate omega-3 PUFA intake, such as 2 g/die, during critical windows of immune maturation may represent a valuable strategy for the primary prevention of allergic diseases. Current evidence most strongly supports supplementation during pregnancy and lactation, particularly in populations with low dietary omega-3 intake or increased allergic risk. Omega-3 supplementation should be considered within a broader multifactorial preventive approach aimed at promoting immune tolerance and reducing the future burden of allergic diseases.

## 1. Introduction: The Epidemiological Context

The rapid increase in the prevalence of type 2 disorders over recent decades has led to the characterization of an “allergy epidemic.” These conditions such as asthma, allergic rhinitis, atopic dermatitis (AD), and food allergies (FAs) now impact a large share of children globally. In Western countries, approximately 30–40% of children are affected by at least one diagnosed allergic condition [1,2]. This trend is particularly evident in industrialized and urbanized regions, suggesting a strong interaction between genetic susceptibility and environmental factors [3,4,5]. Projections indicate a continued rise in allergic conditions. By 2050, more than 50% of the global population is expected to suffer from at least one form of allergy, an exponential rise driven mainly by climate change, air pollution, and alterations in natural habitats [6]. The burden includes direct medical costs, such as hospitalizations and pharmacological treatments, as well as indirect costs related to reduced productivity and long-term morbidity [7,8]. The increasing incidence of FAs alone reflects a broader shift in immune system regulation, likely driven by lifestyle, environmental exposures, and dietary patterns.

In this context, primary prevention has become a critical public health priority. The concept of the “first 1000 days”, spanning from conception to the second year of life, has emerged as a crucial window during which environmental exposures can profoundly influence immune system development and long-term disease risk [9,10]. This period represents a critical window of opportunity during which prenatal, perinatal, and postnatal factors interact to shape long-term health trajectories and susceptibility to disease. Among these, nutritional influences play a particularly pivotal role, as they directly modulate maternal physiology and the immune programming of the fetus and infant [11].

Among dietary components, the balance between pro-inflammatory and anti-inflammatory fatty acids has attracted considerable attention [12,13,14]. Modern Western diets are typically characterized by an excess of Omega-6 polyunsaturated fatty acid (PUFA) relative to Omega-3 PUFA, a shift that may contribute to a pro-inflammatory milieu and increased susceptibility to allergic diseases [15,16,17]. A comprehensive analysis including over 340,000 individuals from 48 countries demonstrated that the majority of populations exhibit suboptimal levels of eicosapentaenoic acid (EPA) and docosahexaenoic acid (DHA), as assessed by the Omega-3 Index (O3I) [18]. Only a limited number of countries, such as Japan, South Korea, and Nordic regions, achieved desirable O3I values (>8%), likely reflecting higher habitual intake of marine-derived omega-3 PUFAs. In contrast, regions including the Middle East, parts of Asia, Africa, and South America displayed particularly low levels (<6%), consistent with low dietary intake of EPA and DHA [18]. Addressing this imbalance through targeted nutritional strategies represents a promising avenue for prevention.

**Literature Search and Quality Assessment**

A scoping literature review and consensus process were conducted to identify and map studies evaluating the role of omega-3 PUFAs in the primary prevention of allergic diseases in children. The literature search was performed using PubMed/MEDLINE, Scopus, and Embase databases up to May 2026. The following keywords and combinations were used: “omega-3”, “polyunsaturated fatty acids”, “PUFA”, “EPA”, “DHA”, “pregnancy”, “lactation”, “allergy prevention”, “asthma”, “wheeze”, “eczema”, “atopic dermatitis”, “food allergy”, and “allergic sensitization”. Randomized controlled trials (RCTs), observational studies, systematic reviews, and meta-analyses published in English were considered. Priority was given to studies evaluating prenatal and early-life exposure to omega-3 PUFAs and clinically relevant allergic outcomes in offspring. The literature search and study selection were conducted by A.K., A.M., and G.C., while C.I. independently verified the included studies and the extracted data.

To improve transparency, eligibility was defined *a priori*. Included records were peer-reviewed English-language RCTs, prospective observational studies, systematic reviews, and meta-analyses addressing prenatal and lactational exposure to omega-3 PUFAs and allergic outcomes in childhood. Studies focused exclusively on adults, animal or in vitro models without translational relevance, non-peer-reviewed reports, or interventions unrelated to EPA/DHA exposure were excluded from the clinical synthesis. Because this article is a SIAIP Position Paper based on a scoping review and consensus process rather than a de novo systematic review, no formal meta-analysis was performed. Given the mixed evidence base, clinical studies were qualitatively appraised using the appropriate Joanna Briggs Institute (JBI) Critical Appraisal Tools according to study design, including RCTs, cohort studies, and analytical cross-sectional studies.

## 2. Characteristics of Polyunsaturated Fatty Acids (PUFA)

PUFAs are essential components of human nutrition, as they cannot be synthesized de novo due to the absence of specific desaturase enzymes. Consequently, they must be obtained through dietary intake. PUFAs are classified according to the position of the first double bond from the methyl (ω) end of the carbon chain, giving rise to two major families: Omega-3 and Omega-6 fatty acids. Omega-3 PUFAs include alpha-linolenic acid (ALA), an essential fatty acid primarily derived from plant sources, and its long-chain derivatives, EPA and DHA, which are mainly found in marine sources [19,20]. Although ALA can be converted into EPA and DHA in the human body, this process is inefficient, with conversion rates typically ranging from 2% to 5%. Furthermore, this conversion is competitively inhibited by high intake of Omega-6 fatty acids, further limiting the availability of biologically active Omega-3 metabolites.

Omega-6 PUFAs include linoleic acid (LA), another essential fatty acid, and arachidonic acid (AA), which is synthesized from LA or obtained directly from animal products. While both Omega-3 and Omega-6 fatty acids are necessary for normal physiological function, their relative balance is critical. Excessive intake of Omega-6 fatty acids, particularly in the context of insufficient Omega-3 intake, has been associated with a pro-inflammatory state that may contribute to the development of chronic inflammatory diseases [21].

Importantly, ALA, EPA, and DHA are not biologically equivalent and exert different physiological activities. ALA mainly acts as a metabolic precursor of long-chain PUFA and shows relatively limited direct biological activity, whereas EPA and DHA represent the biologically active long-chain PUFA. EPA is predominantly involved in anti-inflammatory and immunomodulatory pathways through the generation of less pro-inflammatory eicosanoids and specialized pro-resolving mediators, while DHA mainly exerts structural and neurodevelopmental functions, particularly within neuronal and retinal membranes. Moreover, endogenous conversion of ALA into EPA and especially DHA in humans is limited and highly variable; therefore, no direct quantitative equivalence between ALA, EPA, and DHA can be established [22].

Docosapentaenoic acid (DPA; 22:5 omega-3), the elongated intermediate between EPA and DHA, should also be considered within the omega-3 family. DPA is present in marine foods and immune-cell phospholipids, can be retroconverted to EPA or elongated/desaturated toward DHA, and may generate DPA-derived lipid mediators. Nevertheless, DPA has been far less studied than EPA and DHA in pediatric allergy prevention; at present, its relevance should be regarded as biologically plausible but not sufficient to support DPA-specific clinical recommendations [22,23] (Table 1).

Epidemiological data from the National Health and Nutrition Examination Survey (NHANES) 2007–2016, including 994 children and adolescents, reported that higher omega-6 PUFA intake was associated with increased risk of allergic rhinitis (OR 1.02; 95% CI 1.00–1.04; *p* = 0.03), so exerting a putative pro-inflammatory activity [24]. On the contrary, high omega-3 intake showed no adverse association and was hypothesized to exert anti-inflammatory effects through modulation of gut microbiota and immune pathways [24]. Dietary sources of Omega-3 fatty acids are diverse and include both animal and plant-based options [25] (Table 2). Plant-derived foods, such as flaxseed, chia seeds, walnuts, and vegetable oils, predominantly provide ALA, whereas marine foods mainly contain the biologically active long-chain omega-3 fatty acids EPA and DHA. Fatty fish, including salmon, mackerel, herring, anchovies, sardines, and trout, represent the richest dietary sources of EPA and DHA and are therefore widely recommended, particularly during pregnancy and lactation. From a practical nutritional perspective, marine foods may represent a more feasible strategy for achieving clinically relevant long-chain omega-3 intake in everyday dietary practice. Although plant-derived foods such as flaxseed or chia seeds contain high concentrations of ALA, their conversion into EPA and DHA in humans remains limited. In contrast, a standard portion of fatty fish (approximately 100–150 g of salmon, mackerel, herring, or sardines) may directly provide 1–3 g of highly bioavailable EPA and DHA, which are the biologically active long-chain omega-3 fatty acids most strongly associated with immunomodulatory effects. Consequently, direct dietary intake of long-chain omega-3 fatty acids remains clinically relevant. In addition, edible marine algae and algae-derived products are increasingly recognized as alternative dietary sources of omega-3 fatty acids, particularly for vegetarian and vegan populations [21,26,27,28].

Importantly, the fatty acid composition of fish strongly reflects the composition of the feed administered during aquaculture [29]. Wild fatty fish naturally feed on marine algae and plankton, which represent the primary source of EPA and DHA within the marine food chain [30]. In contrast, farmed fish are increasingly fed with terrestrial vegetable oils, leading to a progressive reduction in long-chain omega-3 fatty acids and a parallel increase in omega-6 fatty acids in fish fillets [31,32]. Wild salmon has been reported to contain approximately 10-fold higher omega-3 than omega-6 levels, whereas in farmed salmon this ratio has progressively declined over recent decades and may even become reversed in some products [33,34]. Furthermore, cooking methods may also influence the final EPA and DHA content of fish products, although several studies suggest that common culinary treatments such as baking, frying, or broiling generally preserve substantial amounts of long-chain omega-3 fatty acids (Table 3).

Environmental contaminant profiles may differ between wild and farmed fish according to marine ecosystem pollution, feed composition, and farming practices. Some recent studies reported higher concentrations of dioxins, polychlorinated biphenyls (PCBs), arsenic, and mercury in wild Atlantic salmon compared with farmed salmon [31,32]. This difference is likely explained by the distinct dietary exposure of wild fish within marine food chains. Wild salmon continuously feed on smaller marine fish, crustaceans, plankton, and other organisms that naturally bioaccumulate persistent organic pollutants and heavy metals from the ocean ecosystem. Through biomagnification processes, these contaminants progressively concentrate at higher trophic levels. In contrast, modern aquaculture increasingly relies on feeds containing substantial amounts of terrestrial vegetable oils and plant-derived ingredients, partially replacing marine fish oils and fishmeal. Since vegetable ingredients contain substantially lower levels of marine-derived persistent pollutants, farmed salmon generally show lower concentrations of dioxins, PCBs, mercury, and arsenic compared with wild fish [31,32]. However, this replacement of marine ingredients also contributes to a reduction in the relative proportion of long-chain omega-3 fatty acids and to an increase in omega-6 fatty acids within farmed salmon fillets (Table 4). However, contaminant concentrations in both wild and farmed salmon generally remain below current European safety limits that set maximum levels for dioxins and dioxin-like PCBs in fish muscle at 650 pg WHO-TEQ/100 g wet weight, for the sum of indicator non-dioxin-like PCBs at 7500 ng/100 g wet weight, and for mercury at 50 µg/100 g wet weight in most fish species, including salmon [35].

## 3. Mechanisms of Immunomodulation

The protective role of PUFAs in allergic disease prevention is mediated through multiple, interconnected biological mechanisms that influence immune function, inflammation, and tissue integrity.

One important mechanism involves the role of long-chain PUFAs as substrates for bioactive lipid mediators. After incorporation into cell membrane phospholipids, fatty acids such as AA, EPA, and DHA may be mobilized through phospholipase A2-dependent pathways; however, cytosolic phospholipase A2 alpha preferentially hydrolyses arachidonate-containing phospholipids, and efficient EPA/DHA release is cell- and context-dependent. AA, an omega-6 fatty acid, is converted into 2-series prostaglandins, thromboxanes, and 4-series leukotrienes, which are generally pro-inflammatory and may contribute to vasodilation and allergic responses. EPA gives rise to mediators that are often less inflammatory than AA-derived eicosanoids, while EPA and DHA can act as precursors of specialized pro-resolving mediators (SPMs), including resolvins, protectins, and maresins [23]. These pathways are biologically compelling, but SPM detection, quantification, and physiological relevance in human samples remain technically debated. Therefore, SPM-related mechanisms should be presented as plausible contributors to immune regulation rather than as definitive proof of clinical efficacy. In parallel, omega-3 PUFAs may influence T-helper cell polarization, regulatory immune pathways, IgE responses, mast-cell activation, and eosinophilic inflammation, but the magnitude and durability of these effects in children remain dependent on timing, dose, baseline omega-3 status, and disease phenotype [36,37,38,39].

Another key mechanism relates to the modulation of the gut microbiota. A higher Omega-3 status has been associated with increased diversity and abundance of beneficial microbial species, which play a crucial role in immune system maturation. The gut microbiota influences the development of immune tolerance, and dysbiosis has been linked to an increased risk of allergic diseases. Furthermore, a higher Omega-3 index has been associated with a greater abundance of beneficial intestinal bacteria and reduced inflammatory status, particularly when accompanied by a lower intake of Omega-6 fatty acids such as arachidonic acid [40]. In addition, maternal Omega-3 intake during pregnancy and lactation affects the composition of human milk oligosaccharides (HMOs), which serve as prebiotics that shape the infant gut microbiome and support immune development [41].

**Figure 1:**
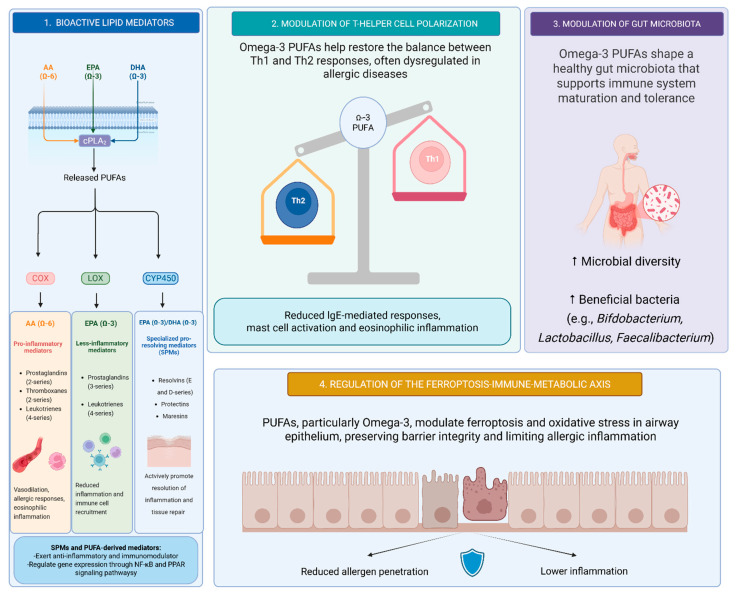
Mechanisms through which omega-3 PUFAs may contribute to the primary prevention of allergic diseases. Omega-3 long-chain PUFAs, particularly EPA and DHA, may modulate allergic disease risk through interconnected pathways, including lipid-mediator production, Th1/Th2 immune balance, epithelial barrier integrity, gut microbiota modulation and oxidative-stress/ferroptosis-related signalling. Abbreviations: PUFA, polyunsaturated fatty acid; EPA, eicosapentaenoic acid; DHA, docosahexaenoic acid; SPMs, specialized pro-resolving mediators; COX, cyclooxygenase, LOX, lipoxygenase; Th1/Th2: T-helper type 1/type 2 balance; HMOs: human milk oligosaccharides; NF-kB: nuclear factor kappa B; PPAR: peroxisome proliferator-activated receptor. The SPM and ferroptosis pathways are promising but remain subject to methodological and translational uncertainty. Created in BioRender. Klain, A. (2026) https://BioRender.com/hzlsvyr.

Emerging research has also highlighted a possible role of PUFAs in the regulation of the ferroptosis-immune-metabolic axis. Ferroptosis is a form of regulated cell death associated with lipid peroxidation and oxidative stress, and dysregulation of this pathway has been implicated in asthma pathogenesis. At present, however, the connection between omega-3 exposure, ferroptosis control, and allergic disease prevention remains largely exploratory and is supported mainly by mechanistic or disease-pathogenesis studies rather than by prevention trials. This pathway should therefore be framed as a hypothesis-generating mechanism that may help explain epithelial barrier protection and oxidative-stress modulation, rather than as an established causal route for preventing allergic disease [42] (Table 5, Figure 1).

## 4. Maternal Omega-3 PUFA Exposure and Allergic Disease Prevention

A growing body of evidence from RCTs, meta-analyses, and observational studies has examined the role of PUFA supplementation in the prevention of allergic diseases. In interpreting these studies, greater weight should be given to adequately powered, blinded RCTs and systematic reviews, whereas observational associations should be considered supportive but vulnerable to residual confounding. This hierarchy of interpretation was aligned with a study-design-specific JBI appraisal summarized in the clinical evidence tables.

Among the most influential studies is the Copenhagen Prospective Studies on Asthma in Childhood (COPSAC2010), a large double-blind, RCT involving 736 pregnant women, of whom 695 offspring were included in the final analysis. Participants were randomized at 24 weeks of gestation to receive 2.4 g/day of EPA and DHA or placebo (olive oil) until one week postpartum, and children were followed prospectively for up to 5 years with detailed clinical phenotyping. The primary outcome was persistent wheeze or asthma. The results demonstrated a significantly reduced risk in the supplementation group (16.9%) compared to the control group (23.7%), corresponding to a hazard ratio of 0.69 (95% CI 0.49–0.97; *p* = 0.035) and a relative risk reduction of approximately 30%. This protective effect remained consistent during extended follow-up to 5 years (hazard ratio 0.68; 95% CI 0.49–0.95) and was even more pronounced in children of mothers with low baseline EPA and DHA levels, where the risk was reduced from 34.1% to 17.5% (hazard ratio 0.46; 95% CI 0.25–0.83), corresponding to a relative reduction of over 50%. In addition, supplementation was associated with a lower incidence of lower respiratory tract infections (31.7% vs. 39.1%; hazard ratio 0.75; 95% CI 0.58–0.98), although no significant effects were observed for allergic sensitization or eczema [43].

A more recent RCT investigated the effects of maternal supplementation with omega-3 PUFAs and the probiotic *Limosilactobacillus reuteri* on HMOs and immune-related factors in breast milk. The study included 136 mother-infant pairs and was conducted as part of an allergy prevention trial, with supplementation administered from mid-pregnancy until 3 months postpartum. Breast milk samples were collected at different time points, including colostrum and mature milk (3 months postpartum), and analyzed for 14 major HMOs. The results showed that maternal omega-3 PUFA supplementation did not significantly alter total HMO concentrations but was associated with a reduction in HMO diversity over time. In contrast, probiotic supplementation alone did not significantly affect HMO composition. Importantly, maternal allergy status was found to influence milk composition, as allergic mothers exhibited significantly lower levels of several HMOs, particularly fucosylated and sialylated structures, compared to non-allergic mothers. In addition, the study identified significant correlations between HMOs and secretory immunoglobulin A (SIgA) levels in breast milk, particularly for fucosylated structures such as lactodifucotetraose (LDFT) and Lacto-N-difucohexaose (LNDFH) (*p* < 0.0001), observed in both colostrum and mature milk. SIgA levels were also positively associated with total and fucosylated HMOs, while weak negative correlations were found with sialylated HMOs, suggesting a functional interaction between these components in shaping infant immune development. This supports the concept of a gut–breast–immune axis through which maternal diet can affect early-life immune programming and potentially influence the risk of allergic disease [44]. In parallel, evidence from a dose–response meta-analysis of RCTs including 3037 mother–infant pairs demonstrated that prenatal supplementation with long-chain omega-3 PUFA (omega-3 LCPUFAs) does not significantly reduce asthma/wheeze risk overall (RR 0.93; 95% CI 0.82–1.04), but shows significant protective effects in specific conditions, particularly at doses ≥1200 mg/day (RR 0.69; 95% CI 0.55–0.88) and when supplementation extends from pregnancy into lactation (RR 0.69; 95% CI 0.51–0.95). Notably, a linear dose–response relationship was observed, with an estimated 2% reduction in risk for every 100 mg/day increase in omega-3 LCPUFA intake [45].

In the DOMInO trial follow-up, involving 706 infants at high hereditary risk of allergic disease, maternal supplementation with 900 mg/day of omega-3 LCPUFAs (800 mg DHA + 100 mg EPA) from 21 weeks’ gestation until delivery did not significantly reduce overall IgE-mediated allergic disease at 1 year (9% vs. 13%; adjusted RR 0.70; 95% CI 0.45–1.09), although a reduction in atopic eczema (7% vs. 12%; RR 0.64) and egg sensitization (9% vs. 15%; RR 0.62; 95% CI 0.41–0.93) was observed [46]. Similarly, longer-term follow-up of the same cohort up to 3 years confirmed no significant reduction in overall IgE-associated allergic disease (17.3% vs. 22.6%; adjusted RR 0.78; 95% CI 0.58–1.06), although trends toward lower eczema prevalence persisted [47].

More recently, Chen et al. provided further mechanistic insight into the relationship between prenatal omega-3 exposure and childhood respiratory outcomes by investigating the role of maternal 12-hydroxyeicosatetraenoic acid (12-HETE), a bioactive lipid mediator involved in immune regulation and alveolar macrophage maturation. Using data from the COPSAC2010 and VDAART birth cohorts, the authors demonstrated that undetectable maternal 12-HETE levels during pregnancy were associated with an increased risk of childhood asthma and respiratory infections, as well as alterations in infant airway microbiota composition and mucosal immune profiles. Notably, the protective effect of prenatal omega-3 LCPUFA exposure appeared to be modified by maternal 12-HETE status. In the COPSAC2010 RCT, omega-3 supplementation significantly reduced the risk of childhood asthma only among offspring of mothers with detectable 12-HETE levels (adjusted HR 0.42; 95% CI 0.22–0.86), whereas no significant effect was observed in those with undetectable levels. Comparable findings were replicated in the independent VDAART cohort using both dietary assessments and circulating biomarkers of maternal omega-3 LCPUFA intake (Table 6) [48]. Evidence from meta-analyses investigating the role of omega-3 PUFAs in allergy prevention has yielded heterogeneous but informative results. A Cochrane systematic review including eight RCTs involving 3366 women and 3175 children found that maternal omega-3 long-chain PUFA supplementation during pregnancy and/or lactation was associated with a significant reduction in IgE-mediated allergic disease in early childhood (12–36 months: RR 0.66; 95% CI 0.44–0.98), although this effect was not sustained beyond 36 months (RR 0.86; 95% CI 0.61–1.20), and no consistent reduction was observed for asthma or wheeze outcomes [49]. A systematic review and meta-analysis including 13 cohort studies and 5 RCTs reported that increased maternal intake of omega-3 LCPUFAs during pregnancy was associated with a reduced risk of early allergic outcomes, including atopic eczema (RR 0.53; 95% CI 0.35–0.81), any sensitization (RR 0.68; 95% CI 0.52–0.89), egg sensitization (RR 0.55; 95% CI 0.39–0.76), and food sensitization (RR 0.59; 95% CI 0.46–0.76) during the first year of life, although heterogeneity across trials limited definitive conclusions [50].

Consistently, a more recent systematic review from the European Academy of Allergy and Clinical Immunology, including 95 studies (17 RCTs and 78 observational studies), reported a trend toward a protective effect of prenatal omega-3 supplementation on asthma and wheeze in offspring (pooled OR 0.70; 95% CI 0.45–1.08), although this did not reach statistical significance, and no consistent effect was observed for eczema, FA, or allergic sensitization [51]. In contrast to supplementation trials, dietary exposure to fish during infancy appears to show more consistent protective effects. A systematic review and meta-analysis including 13 prospective cohort studies and one RCT reported that fish intake during the first year of life was associated with a significant reduction in the risk of eczema (RR 0.61; 95% CI 0.47–0.80) and allergic rhinitis (RR 0.54; 95% CI 0.36–0.81), whereas maternal fish intake during pregnancy was not associated with a reduced risk of allergic outcomes [52].

Evidence from observational cohort studies further supports a potential protective role of prenatal polyunsaturated fatty acids in allergic disease development. In the study by Maslova et al., conducted within the Project Viva cohort (n = 1356 mother-child pairs), maternal plasma levels of omega-3 and omega-6 fatty acids were measured during the second trimester, and children were followed longitudinally from early childhood (median age 3.3 years) to mid-childhood (median age 7.7 years). The authors reported that higher maternal concentrations of long-chain omega-3 PUFAs, particularly EPA, were associated with a reduced risk of allergic sensitization and current asthma in mid-childhood. Specifically, children in the highest quartile of maternal EPA levels had a significantly lower risk of allergic sensitization (adjusted OR 0.56; 95% CI 0.32–0.97) and current asthma (adjusted OR 0.55; 95% CI 0.31–0.98) compared to those in the lowest quartile. Notably, these associations were more evident in mid-childhood than in early childhood, suggesting a potential long-term effect of prenatal fatty acid exposure on immune development. In contrast, higher maternal fish and shellfish intake was unexpectedly associated with an increased risk of allergic sensitization (OR 1.65; 95% CI 1.07–2.52), highlighting the complexity of dietary exposures and the potential influence of food-specific factors [53]. According to a JBI-oriented qualitative appraisal for cohort studies, this evidence can be considered of moderate methodological quality, as it derives from a prospective cohort with biomarker-based exposure assessment and long follow-up, although residual confounding, non-randomized exposure, dietary complexity, and possible attrition bias limit causal interpretation.

## 5. Limitations

Despite the growing body of evidence supporting a potential role of omega-3 PUFAs in the prevention of allergic diseases, several limitations should be acknowledged. First, this SIAIP position paper did not perform a de novo systematic review or meta-analysis; methodological quality and risk of bias were assessed qualitatively using study-design-specific JBI Critical Appraisal Tools rather than through a single quantitative grading system. Second, available studies are highly heterogeneous with regard to study design, population characteristics, family history of allergy, timing and duration of supplementation, EPA/DHA dose, baseline dietary intake, formulation, comparator oils, and assessed allergic outcomes. Third, many RCTs were not primarily powered for allergy-related endpoints, and follow-up often ended before allergic phenotypes were fully established. Fourth, observational studies are susceptible to residual confounding by diet quality, breastfeeding, socioeconomic status, environmental exposures, and health behaviours.

## 6. Current Recommendations

According to the European Food Safety Authority (EFSA), age-specific dietary reference values for PUFAs highlight the critical importance of adequate omega-3 intake throughout early life and during pregnancy. In infancy, a minimum intake of DHA of 100 mg/day is recommended starting from approximately 7 months of age. During childhood, omega-3 requirements increase, with DHA maintained at 100 mg/day up to 2 years of age and a combined intake of EPA and DHA of 250 mg/day recommended thereafter, alongside an adequate intake of ALA corresponding to approximately 0.5% of total energy intake from the age of 2 years. In pregnant and lactating women, requirements are further elevated, with a recommended intake of 250 mg/day of EPA + DHA plus an additional 100–200 mg/day of DHA to support fetal and neonatal development [54].

Current dietary recommendations further support an adequate intake of marine-derived omega-3 fatty acids during pregnancy and across the life course. A recent scoping review informing the Nordic Nutrition Recommendations (NNR2023) highlighted that most Nordic countries advise a fish intake of approximately 300–450 g/week (corresponding to 2–3 servings), with a substantial proportion derived from fatty fish rich in EPA and DHA, reflecting the established health benefits of long-chain omega-3 PUFAs [55]. Despite these recommendations, epidemiological data indicate that actual intake of omega-3 PUFAs is frequently below recommended levels, particularly in vulnerable populations such as pregnant or breastfeeding women. This inadequacy is largely attributed to dietary patterns characteristic of Western societies, which are marked by a disproportionately high intake of omega-6 fatty acids relative to omega-3, resulting in an unfavourable omega-6/omega-3 ratio that may promote pro-allergic immune responses and contribute to the increasing prevalence of allergic diseases.

## 7. Italian Society of Pediatric Allergy and Immunology (SIAIP) Position Statement

Although the available evidence is heterogeneous, a consistent but not definitive body of data suggests that adequate maternal intake of omega-3 LCPUFAs during pregnancy and lactation may contribute to a reduced risk of selected allergic outcomes in offspring, particularly wheezing and asthma, with less consistent evidence for allergic sensitisation, eczema, and FA. Importantly, the observed effects appear to be more closely related to long-chain derivatives, EPA and DHA, than to shorter-chain precursors such as ALA, whose conversion is limited. Clinical trials and meta-analyses suggest that any protective effect is more evident when supplementation provides sufficiently high amounts of EPA and DHA (2.4–3.7 g/die), when baseline maternal omega-3 status is low, or when hereditary risk is increased. The SIAIP Primary and Secondary Prevention of Allergic Diseases Commission therefore recommends assessing habitual fish intake, baseline dietary omega-3 intake, allergic risk, and possible contraindications. Adequate intake of long-chain omega-3 PUFAs, particularly EPA and DHA, should be promoted through regular consumption of low-contaminant fatty fish or other marine sources when acceptable and feasible. However, achieving an adequate daily intake of long-chain omega-3 fatty acids may be difficult through a conventional diet alone. Therefore, when dietary intake is insufficient, targeted supplementation with purified, quality-controlled omega-3 preparations should be considered, especially in women with low omega-3 intake/status or increased familial risk of allergic disease. A daily intake of approximately 2 g of combined EPA and DHA may be considered as part of a preventive nutritional strategy, particularly in high-risk populations. From a safety perspective, available evidence indicates that long-chain omega-3 PUFA supplementation is generally well tolerated, and no significant adverse effects have been consistently reported at the doses evaluated in clinical studies, including intakes in the range of 2–3 g/day. These levels are considered safe within the context of dietary and supplemental intake in otherwise healthy pregnant and lactating women, although clinical judgment remains appropriate in individual cases.

Importantly, not all fish products or omega-3 nutraceutical formulations provide equivalent nutritional and biological value. In clinical practice, considerable variability exists among commercially available omega-3 supplements with regard to EPA and DHA concentration, purity, oxidation status, and pharmaceutical quality. Some products marketed as ‘omega-3 supplements’ may contain only minimal amounts of biologically active long-chain omega-3 fatty acids, providing quantities that are substantially below those used in clinical trials investigating allergy prevention and immunomodulation. Therefore, clinicians should carefully evaluate the actual EPA and DHA content per dose rather than relying exclusively on the total amount of fish oil declared on the label. Preference should be given to formulations characterized by adequate concentrations of EPA and DHA, high oxidative stability, low oxidation indices, and rigorous purification standards aimed at reducing environmental contaminants such as heavy metals, dioxins, and PCBs. In addition, naturally occurring triglyceride-based fish oils may better preserve the physiological structural complexity of marine fatty acids compared with highly concentrated chemically modified formulations, although additional comparative studies are still needed. The source of omega-3 fatty acids also deserves consideration. Wild fatty fish generally display a more favourable omega-3/omega-6 ratio compared with farmed fish, whereas algae-derived formulations may represent a valuable alternative for vegetarian or vegan individuals. Sustainability and responsible sourcing from certified fisheries or controlled algal cultivation should also be considered important components in the selection of marine-derived omega-3 products.

However, omega-3 supplementation should not be viewed as an isolated intervention, but rather as part of a broader allergy-prevention approach. This includes adequate maternal nutrition, avoidance of tobacco smoke exposure, promotion of breastfeeding, when possible, timely introduction of complementary foods, including fish and other allergenic foods, and support for a lifestyle that favours immune tolerance during the first 1000 days of life [56,57,58,59,60].

## 8. Conclusions

Omega-3 PUFAs represent a biologically plausible and clinically relevant nutritional strategy within the primary prevention of allergic diseases. The most consistent clinical signal concerns maternal EPA/DHA exposure during pregnancy and lactation and later respiratory outcomes, especially wheeze/asthma, in women with low baseline omega-3 status or offspring at increased allergic risk. Evidence for eczema, FA and allergic sensitization remains more heterogeneous. In conclusion, when dietary omega-3 intake is inadequate, targeted supplementation with purified, quality-controlled omega-3 products may be considered, particularly for women with low omega-3 intake or status, or with a family history of allergic disease, approximately 2 g/day in selected high-risk contexts and under clinical supervision.

## Figures and Tables

**Table 1:** Structural classification of the main omega-3 and omega-6 PUFA families and their long-chain derivatives. ALA: alpha-linolenic acid; EPA: eicosapentaenoic acid; DPA: docosapentaenoic acid; DHA: docosahexaenoic acid; LA: linoleic acid; GLA: gamma-linolenic acid; DGLA: dihomo-gamma-linolenic acid; AA: arachidonic acid.

PUFA Family	Biosynthetic Sequence and Principal Derivatives
Omega-3	ALA (18:3 omega-3) → EPA (20:5 omega-3) → DPA (22:5 omega-3) → DHA (22:6 omega-3)
Omega-6	LA (18:2 omega-6) → GLA (18:3 omega-6) → DGLA (20:3 omega-6) → AA (20:4 omega-6)

**Table 2:** Approximate content of ALA, EPA, and DHA in selected dietary sources, expressed per 100 g of edible product. Plant-derived foods predominantly provide ALA, whereas fish and seafood mainly provide EPA and DHA. Values were adapted and converted from serving-based data reported by the National Institutes of Health Office of Dietary Supplements and USDA FoodData Central. Values may vary according to species, geographical origin, farming conditions, and processing methods. Modified from NIH Office of Dietary Supplements [25].

Food Source	ALA (g/100 g)	EPA (g/100 g)	DHA (g/100 g)
***Vegetable oils, seeds and nuts***			
**Flaxseed oil**	53.4	0	0
**Chia seeds**	17.8	0	0
**Flaxseed, whole**	22.5	0	0
**English walnuts**	9.1	0	0
**Black walnuts**	2.7	0	0
**Canola oil**	9.1	0	0
**Soybean oil**	6.8	0	0
**Mayonnaise**	4.9	0	0
***Legumes and plant-derived foods***			
**Edamame**	0.6	0	0
**Refried beans**	0.4	0	0
**Kidney beans**	0.2	0	0
**Baked beans**	0.1	0	0
**Whole wheat bread**	0.1	0	0
***Fish and seafood***			
**Atlantic salmon, farmed**	0	0.87	1.83
**Atlantic salmon, wild**	0	0.52	1.79
**Herring, Atlantic**	0	1.13	1.38
**Sardines, canned**	0	0.66	1.09
**Atlantic mackerel**	0	0.63	0.87
**Pink salmon, canned**	0.06	0.41	0.93
**Rainbow trout, wild**	0	0.59	0.65
**Sea bass**	0	0.26	0.68
**Tuna, light canned**	0	0.03	0.25
**Tuna, yellowfin**	0	0.01	0.13
**Pacific cod**	0	0.06	0.15
**Tilapia**	0.06	0	0.13
**Shrimp**	0	0.18	0.18
**Lobster**	0.06	0.15	0.10
**Oysters**	0.21	0.44	0.34
**Scallops**	0	0.09	0.14
***Animal-derived foods***			
**Egg**	0	0	0.03
**Chicken breast**	0	0.01	0.02
**Ground beef (85% lean)**	0.06	0	0
**Low-fat milk**	0.01	0	0

**Table 3:** EPA + DHA content in selected commonly consumed wild fish from natural ecosystems according to preparation method. Values are expressed uniformly as g/100 g of edible product; daily portions were calculated from the EPA + DHA content required to provide 1 g/day. Modified from Gladyshev MI, Sushchik NN. Biomolecules. 2019 [34].

Fish Species	Preparation Method	EPA + DHA (g/100 g Product)	Daily Portion for 1 g EPA + DHA (g)
Atlantic salmon (*Salmo salar*)	Fried	1.7–4.0	25–59
King salmon (*Oncorhynchus tshawytscha*)	Baked	1.24	81
King salmon (*Oncorhynchus tshawytscha*)	Fried	1.15	87
Pacific herring (*Clupea harengus*)	Canned	1.79	56
Pacific herring (*Clupea harengus*)	Boiled	0.39	256
Pacific herring (*Clupea harengus*)	Fried	0.38	263
Sardine (*Sardina pilchardus*)	Fried	0.88	114
Lake trout (*Salvelinus namaycush*)	Baked	1.24	81
Lake trout (*Salvelinus namaycush*)	Fried	1.24	81
Lake trout (*Salvelinus namaycush*)	Boiled	1.23	81
Brown trout (*Salmo trutta*)	Boiled	0.57	175
Brown trout (*Salmo trutta*)	Fried	0.41	244
Atlantic cod (*Gadus morhua*)	Fried	0.22–0.41	244–455
Atlantic cod (*Gadus morhua*)	Boiled	0.24	417
Spanish mackerel (*Scomberomorus commerson*)	Fried	0.39	256
Common carp (*Cyprinus carpio*)	Fried	0.10	1000
Common carp (*Cyprinus carpio*)	Baked	0.07	1429
Common carp (*Cyprinus carpio*)	Boiled	0.05	2000

**Table 4:** Comparison of lipid composition and selected environmental contaminants between wild and farmed Atlantic salmon. Values are expressed per 100 g of edible product unless otherwise specified. EPA: eicosapentaenoic acid; DHA: docosahexaenoic acid; PUFA: polyunsaturated fatty acids; FA: fatty acids; LA: linoleic acid; ALA: alpha-linolenic acid; DL-PCBs: dioxin-like polychlorinated biphenyls; TEQ: toxic equivalents.

Parameter	Wild Salmon	Farmed Salmon
Total fat	6 g/100 g	17.9 g/100 g
EPA	0.4 g/100 g	0.5 g/100 g
DHA	0.8 g/100 g	0.9 g/100 g
Total EPA + DHA	1.2 g/100 g	1.4 g/100 g
Omega-3 content on total fat (%)	20%	7.8%
LA	0.1 g/100 g	2.5 g/100 g
ALA	0.1 g/100 g	1.8 g/100 g
Dioxins	53 pg TEQ/100 g	23–52 pg TEQ/100 g
DL-PCBs	95 pg TEQ/100 g	29 pg TEQ/100 g
Mercury	5.63 µg/100 g	1.5 µg/100 g

**Table 5:** Main biological mechanisms through which PUFAs, particularly omega-3 fatty acids, may contribute to the prevention and modulation of allergic diseases. The pathways should be interpreted as mechanistically plausible and not uniformly proven in human allergy-prevention trials. Abbreviations: AA: arachidonic acid; HMOs: human milk oligosaccharides; SPMs: specialized pro-resolving mediators.

Biological Mechanism	Role of PUFAs/Omega-3 Fatty Acids	Potential Effect on Allergic Diseases
**Production of bioactive lipid mediators**	EPA and DHA can be converted into less inflammatory mediators and specialized pro-resolving mediators, including resolvins, protectins, and maresins; human quantification and physiological relevance remain debated	Reduction in inflammation and promotion of inflammatory resolution
**Competition with arachidonic acid**	Omega-3 fatty acids reduce the production of AA-derived pro-inflammatory prostaglandins and leukotrienes	Decreased vasodilation, inflammation, and allergic responses
**Regulation of NF-κB and PPAR pathways**	SPMs may modulate transcriptional pathways involved in inflammation, although clinical translation remains uncertain	Anti-inflammatory and immunomodulatory effects
**Modulation of Th1/Th2 balance**	Omega-3 fatty acids help restore the balance between Th1 and Th2 immune responses	Reduction in Th2-skewed allergic inflammation
**Reduction in Th2 cytokines**	Decreased production of IL-4, IL-5, and IL-13	Reduced IgE-mediated responses, mast cell activation, and eosinophilic inflammation
**Enhancement of regulatory immune pathways**	Promotion of anti-inflammatory and tolerogenic immune responses	Improved control of allergic manifestations
**Gut microbiota modulation**	Increased diversity and abundance of beneficial intestinal bacteria	Improved immune maturation and immune tolerance
**Reduction in dysbiosis**	Improved Omega-3/Omega-6 balance	Reduced inflammatory status and lower allergy risk
**Influence on human milk oligosaccharides (HMOs)**	Maternal Omega-3 intake modifies HMO composition during pregnancy and lactation	Support of infant gut microbiome development and immune maturation
**Regulation of the ferroptosis-immune-metabolic axis**	Omega-3 fatty acids may influence lipid peroxidation and oxidative stress in selected contexts	Hypothesis-generating protection of airway epithelium and attenuation of inflammation
**Maintenance of epithelial barrier integrity**	Reduction in oxidative epithelial damage	Decreased penetration of environmental allergens

**Table 6:** Summary of RCTs, meta-analysis and clinical studies investigating prenatal and early-life omega-3 PUFA exposure, supplementation dosage, and allergic disease outcomes in offspring. EPA: eicosapentaenoic acid; DHA: docosahexaenoic acid; PUFA: polyunsaturated fatty acid; LCPUFA: long-chain polyunsaturated fatty acid; HMO: human milk oligosaccharide; SIgA: secretory immunoglobulin A; AD: atopic dermatitis; IgE: immunoglobulin E; HR: hazard ratio; COPSAC2010: Copenhagen Prospective Studies on Asthma in Childhood 2010; VDAART: Vitamin D Antenatal Asthma Reduction Trial; 12-HETE: 12-hydroxyeicosatetraenoic acid. Methodological quality and risk of bias were qualitatively appraised using JBI Critical Appraisal Tools selected according to study design.

Reference	Population/Timing of Exposure	Omega-3 Supplementation (Dose)	Main Findings	Methodological Quality/Risk of Bias
[43]	Pregnant women (prenatal exposure); supplementation from 24 weeks’ gestation to 1 week postpartum	2.4 g/day EPA + DHA	Reduced risk of persistent wheeze/asthma in offspring (HR 0.69); stronger effect in mothers with low baseline EPA/DHA (HR 0.46)	High quality/low-to-moderate risk of bias: large double-blind RCT with prospective phenotyping; residual uncertainty for non-respiratory outcomes.
[44]	Pregnant and lactating women; from mid-pregnancy to 3 months postpartum	3.84 g/day omega-3 PUFAs (6 capsules/day; each capsule containing 640 mg omega-3 PUFAs: 35% EPA and 25% DHA)	Modulation of HMO diversity and correlations with SIgA levels in breast milk	Moderate quality/indirect evidence: randomized design, but HMO/SIgA endpoints are mechanistic rather than direct allergy outcomes.
[45]	Pregnant women; some studies extended into lactation	Significant effects observed at 1200 mg/day or higher omega-3 LCPUFAs	No overall reduction in asthma/wheeze risk, but significant protection at higher doses and with prolonged supplementation	Moderate-to-high quality/heterogeneous risk: dose–response meta-analysis of RCTs; certainty limited by variable dose, timing, and outcome definitions.
[46]	Pregnant women carrying infants at high allergic risk; from 21 weeks’ gestation until delivery	900 mg/day omega-3 LCPUFAs (800 mg DHA + 100 mg EPA)	Reduced AD and egg sensitization, but no significant reduction in overall allergic disease	Moderate quality/some risk of bias: RCT follow-up; allergy endpoints partly subgroup or secondary, and overall allergic disease was not significantly reduced.
[47]	Prenatal exposure; follow-up of offspring up to 3 years	900 mg/day omega-3 LCPUFAs	No significant reduction in overall IgE-mediated allergic disease; persistent trend toward lower eczema prevalence	Moderate quality/follow-up limitations: RCT cohort follow-up with attrition and non-significant overall IgE-mediated allergic disease outcome.
[48]	Pregnant women from COPSAC2010 and VDAART cohorts	2.4 g/day omega-3 LCPUFA (fish oil) containing EPA and DHA during the third trimester of pregnancy.	Protective effect against asthma observed only in offspring of mothers with detectable 12-HETE levels	Moderate quality/risk of residual confounding: combined RCT and cohort biomarker analyses; response-modification hypothesis requires confirmation.
[49]	Systematic review of 8 RCTs; maternal supplementation during pregnancy and/or lactation	Maternal omega-3 LCPUFA supplementation	Reduced IgE-mediated allergic disease at 12–36 months, but effect not sustained beyond 36 months; no consistent reduction in asthma/wheeze	High methodological quality/heterogeneous evidence: Cochrane review of RCTs; certainty limited by outcome heterogeneity, timing, follow-up duration, and inconsistent persistence of benefit
[50]	Systematic review/meta-analysis including 13 cohort studies and 5 RCTs	Increased maternal omega-3 LCPUFA intake during pregnancy	Reduced early allergic outcomes including eczema, sensitization, egg sensitization, and food sensitization, but heterogeneity limited definitive conclusions	Moderate-to-high quality/mixed-study evidence: includes both RCTs and observational studies; risk of heterogeneity and residual confounding limits causal interpretation
[51]	EAACI systematic review including 95 studies	Prenatal omega-3 supplementation and dietary exposures	Trend toward protection for asthma/wheeze, not statistically significant; no consistent effect for eczema, FA, or allergic sensitization	Moderate-to-high quality/heterogeneous evidence: broad systematic review with many observational studies; conclusions limited by mixed designs, exposure definitions, and outcome variability
[52]	Systematic review/meta-analysis of fish intake during pregnancy or infancy; mainly prospective cohorts plus one RCT	Dietary fish intake, not direct omega-3 supplementation	Fish intake during infancy associated with reduced eczema and allergic rhinitis; maternal fish intake during pregnancy not associated with reduced allergic outcomes	Moderate quality/indirect evidence: fish intake is a dietary proxy rather than purified EPA/DHA supplementation; high heterogeneity and observational designs limit causal inference
[53]	Project Viva prospective cohort; maternal plasma fatty acids measured in second trimester; follow-up to mid-childhood	Maternal plasma omega-3 and omega-6 fatty acid levels; dietary fish/shellfish intake	Higher maternal EPA associated with lower allergic sensitization and current asthma in mid-childhood; higher fish/shellfish intake unexpectedly associated with increased sensitization	Moderate quality/moderate risk of bias: prospective cohort with biomarker-based exposure and long follow-up; non-randomized exposure, residual confounding, dietary complexity, and possible attrition bias limit causal interpretation

## Data Availability

The original contributions presented in this study are included in the article. Further inquiries can be directed to the corresponding author(s).

## Abbreviations

The following abbreviations are used in this manuscript:

AA	Arachidonic Acid
AD	Atopic Dermatitis
ALA	Alpha-Linolenic Acid
cPLA2	Cytosolic Phospholipase A2
ARA	Arachidonic Acid
CAPS	Childhood Asthma Prevention Study
CI	Confidence Interval
COPSAC2010	Copenhagen Prospective Studies on Asthma in Childhood 2010
COX	Cyclooxygenase
DHA	Docosahexaenoic Acid
DPA	Docosapentaenoic Acid
DL-PCBs	Dioxin-Like Polychlorinated Biphenyls
EFSA	European Food Safety Authority
EPA	Eicosapentaenoic Acid
FA	Food Allergy
HMOs	Human Milk Oligosaccharides
HR	Hazard Ratio
IgE	Immunoglobulin E
IL	Interleukin
LA	Linoleic Acid
LCPUFA	Long-Chain Polyunsaturated Fatty Acid
LDFT	Lactodifucotetraose
LNDFH	Lacto-N-Difucohexaose
LOX	Lipoxygenase
NF-κB	Nuclear Factor Kappa B
NHANES	National Health and Nutrition Examination Survey
O3I	Omega-3 Index
OR	Odds Ratio
PCB	Polychlorinated Biphenyl
PPAR	Peroxisome Proliferator-Activated Receptor
PUFA	Polyunsaturated Fatty Acid
RCT	Randomized Controlled Trial
RR	Relative Risk
SIgA	Secretory Immunoglobulin A
SIAIP	Italian Society of Pediatric Allergy and Immunology
SPMs	Specialized Pro-Resolving Mediators
TEQ	Toxic Equivalents
Th	T-helper
USDA	United States Department of Agriculture
VDAART	Vitamin D Antenatal Asthma Reduction Trial
WHO	World Health Organization
